# Ribonucleotide reductases: essential enzymes for bacterial life

**DOI:** 10.3389/fcimb.2014.00052

**Published:** 2014-04-28

**Authors:** Eduard Torrents

**Affiliations:** Bacterial Infections and Antimicrobial Therapies Group, Institute for Bioengineering of CataloniaBarcelona, Spain

**Keywords:** ribonucleotide reductase, evolution, gene regulation, DNA synthesis, NrdR, transcriptional regulation, anaerobiosis

## Abstract

Ribonucleotide reductase (RNR) is a key enzyme that mediates the synthesis of deoxyribonucleotides, the DNA precursors, for DNA synthesis in every living cell. This enzyme converts ribonucleotides to deoxyribonucleotides, the building blocks for DNA replication, and repair. Clearly, RNR enzymes have contributed to the appearance of genetic material that exists today, being essential for the evolution of all organisms on Earth. The strict control of RNR activity and dNTP pool sizes is important, as pool imbalances increase mutation rates, replication anomalies, and genome instability. Thus, RNR activity should be finely regulated allosterically and at the transcriptional level. In this review we examine the distribution, the evolution, and the genetic regulation of bacterial RNRs. Moreover, this enzyme can be considered an ideal target for anti-proliferative compounds designed to inhibit cell replication in eukaryotic cells (cancer cells), parasites, viruses, and bacteria.

## Introduction

**Figure 1:**
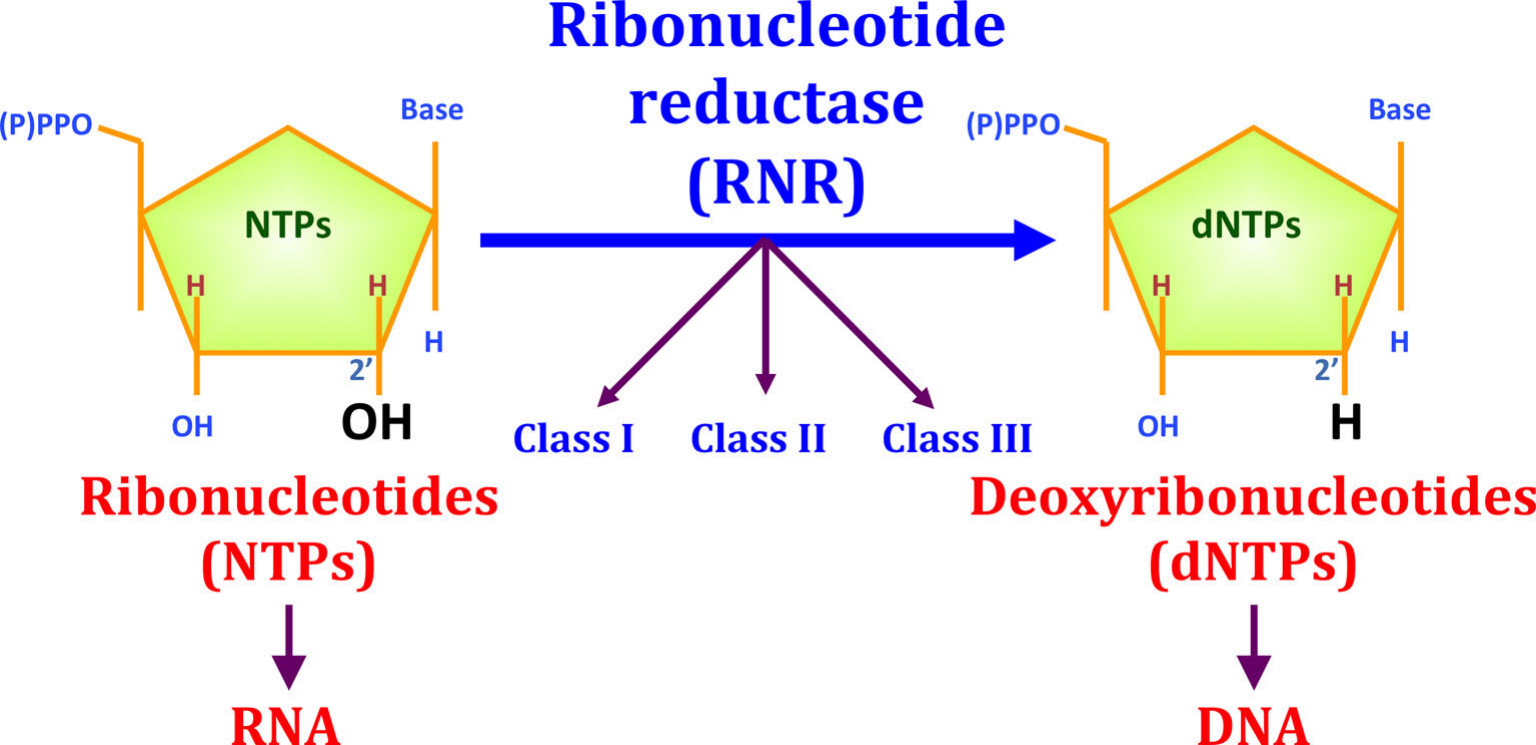
**The reduction of ribonucleotides to deoxyribonucleotides by RNR**. Three different RNR classes (I, II, and III) have been described for this enzyme family. RNR is important for evolution, as this enzyme played an important role during the transition from an RNA to a DNA world. RNR enzymes catalyze the reduction of the ribose C2′-OH to C2′-H.

All organisms require active DNA synthesis prior to cell division. A first prerequisite for DNA synthesis is the balanced supply of the different deoxyribonucleotide triphosphates (dNTPs). The only biochemical pathway for *de novo* dNTP synthesis is the reaction catalyzed through the enzyme **ribonucleotide reductase (RNR**), which converts the four ribonucleotides triphosphates (NTPs) into their corresponding dNTPs through the reduction of the C2′-OH bond (see Figure 1).

### Ribonucleotide reductase (RNR): structure and mechanisms

RNR uses radical chemistry to catalyze the reduction of each NTP. How the enzyme generates this radical, the type of cofactor and metal required, the three-dimensional structure of this enzyme complex and the dependence of oxygen are all characteristics that are considered when classifying RNRs. Currently, three different RNR classes have been described (I, II, and III), and class I is further subdivided into Ia, Ib, and Ic (see Table 1). All three RNR classes share a common three-dimensional protein structure at the catalytic subunit and a highly conserved α/β barrel structure in the active site of the enzyme. In addition, the two potential allosteric centers (specificity and activity) are highly conserved among the different RNR classes, although in class Ib, and some class II RNRs activity allosteric site is absent (reviewed in Nordlund and Reichard, 2006; Hofer et al., 2012).

**Table 1:** **Overview of RNR classes**.

	**Class Ia**	**Class Ib**	**Class Ic**	**Class II**	**Class III**
Oxygen dependence	Aerobic	Aerobic	Aerobic	Aerobic/Anaerobic	Anaerobic
Structure	α_2_β_2_/α_6_β_6_	α_2_β_2_	α_2_β_2_	α(α_2_)	α_2_ + β_2_
Gene	*nrdAB*	*nrdHIEF*	*nrdAB*	*nrdJ*	*nrdDG*
Radical	Tyr… Cys	Tyr… Cys	Phe… Cys	AdB12… Cys	AdoMet… Gly… Cys?
Metallocofactor “*in vivo*”	Fe^III^-O-Fe^III^	Mn^III^-O-Mn^III^ Fe^III^-O-Fe^III^	Mn^IV^-O-Fe^III^	Co	Fe^II^-S^II^
Cofactor assembly	YfaE	NrdI	Unknown	Unknown	IscF
Substrate	NDP	NDP	NDP	NDP/NTP	NTP
Reductant	Thioredoxin glutaredoxin	NrdH-redoxin glutaredoxin	Unknown	Thioredoxin	Formate
Number of allosteric sites	2	1	2	1/2	2
ATP inhibition	Yes	No	Yes	Yes/No	Yes
Distribution	Eukaryotes eubacteria archaea bacteriophages virus	Eubacteria	Eubacteria	Archaea eubacteria bacteriophages	Archaea eubacteria bacteriophages

Reduction of the four different nucleotides (ATP/CTP/GTP/TTP) occurs at a single active site in each polypeptide chain, therefore the tight regulation of dNTP levels is important for each dividing cell. Unbalanced dNTP levels could lead to increased mutation rates (Mathews, 2006). Thus, one of the most important aspects of the dNTP supply required for DNA synthesis and repair is the tight regulation of RNRs at different levels, including the allosteric regulation of enzyme activity, transcriptional regulation, and cell cycle-specific proteolysis in mammalian cells.

**Figure 2:**
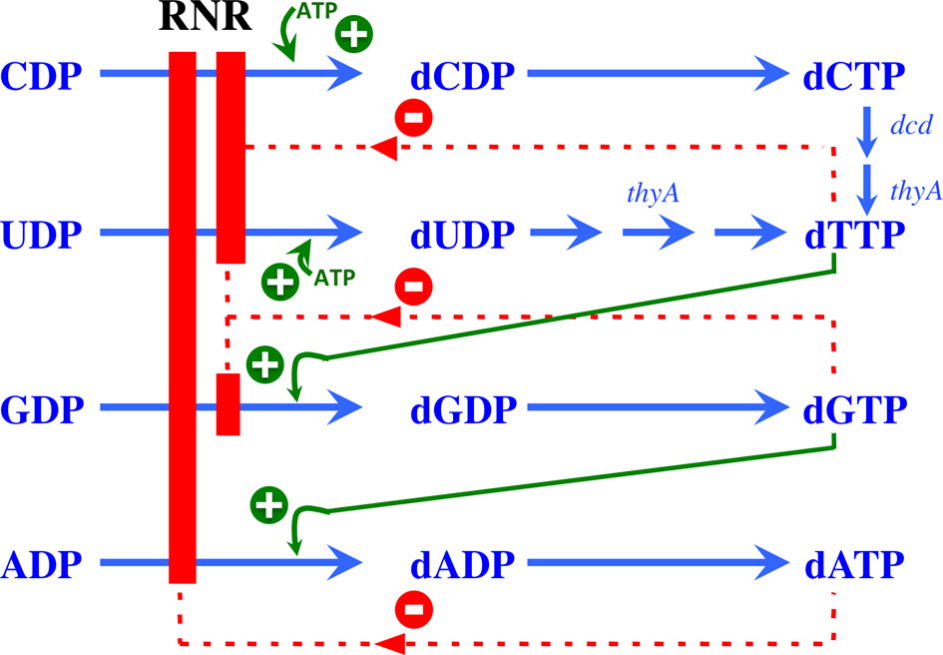
**Allosteric regulation of RNR**. Model showing the allosteric regulation of class Ia RNRs. The binding of ATP at the substrate specificity site activates the enzyme, promoting the reduction of CDP and UDP to dCDP and dUDP, respectively. Once formed, dTTP promotes the reduction of GDP to dGDP, which in turn induces the reduction of ADP to dADP. A high dATP concentration inhibits the overall activity of this enzyme through binding to the allosteric activity site. Green plus symbols indicate stimulation of RNR reduction, and red minus symbols indicate inhibition.

RNR activity is controlled at two different levels: substrate specificity, in which the binding of different nucleotides results in the reduction of each specific NTP at the active site, and enzymatic activity, in which the binding of ATP, or dATP respectively activates or inhibits enzymatic activity (Figure 2). Extensive reviews concerning allosteric regulation at the biochemical level have previously been published (Reichard, 1997; Nordlund and Reichard, 2006; Hofer et al., 2012; Ahmad and Dealwis, 2013), thus this review will focus on the bacterial RNR rather than the eukaryotic RNR.

#### Class I

Class I RNRs are the best-known and most-studied enzymes. These enzymes comprise two homodimeric subunits, namely R1 (or α) and R2 (or β). The α subunit contains the catalytic subunit, containing the active site where nucleotide reduction occurs, and two allosteric sites, involved in the allosteric regulation of substrate specificity and general enzyme activity. The β subunit harbors the metallocofactor essential for the initiation of nucleotide reduction.

The active form of RNR in eukaryotes and prokaryotes comprises two proteins (R1 + R2 or α + β) associated in a dimeric or other oligomeric forms, such as α_n_β_m_ (where n can be 2, 4 or 6 and m 1, 2, 3 or more). Several reviews and studies have previously described the structural basis for the allosteric regulation and cluster assembly of class I RNRs (Ando et al., 2011; Hofer et al., 2012; Ahmad and Dealwis, 2013; Tomter et al., 2013).

Class I RNRs can be further subdivided into class Ia, Ib, and Ic enzymes (see Table 1) based on the type of metal center required to generate the protein radical.

The *nrdAB* genes encode class Ia enzymes, which require a di-iron center (Fe^III^-O-Fe^III^) in the NrdB (β) subunit to generate the tyrosyl radical. The *nrdHIEF* genes encode the class Ib operon, with NrdE and NrdF encoding the α and β subunits, respectively, NrdI encoding a flavodoxin (Cotruvo and Stubbe, 2008; Roca et al., 2008b) and NrdH encoding a glutaredoxin-like protein (Jordan et al., 1997; Crona et al., 2011). NrdI encodes a specific protein involved in the biosynthesis and maintenance of the active metal center, and NrdH is the specific electron donor for the NrdEF enzyme system.

Class Ib RNRs harbor a di-manganese center (Mn^III^-O-Mn^III^) to generate the tyrosyl radical *in vivo*, although this radical can also be generated with a di-ferric center (Fe^III^-O-Fe^III^) (Cotruvo and Stubbe, 2010, 2011, 2012). The catalytic subunit NrdE also differs from that of other class I RNRs because this enzyme lacks the activity site at the N-terminal region of the protein.

Moreover, the *nrdAB* genes encode class Ic RNRs, which is distinguished from class Ia RNRs, as the protein radical is generated through a Mn^IV^-O-Fe^III^ center (Jiang et al., 2007a,b; Dassama et al., 2012).

During catalysis, the radical is formed in the β subunit in all three class I RNRs and subsequently transferred to the large subunit (α) via a long-range radical transfer pathway, generating a thiol radical at the active site of the enzyme, where two cysteines are ultimately responsible for NTP reduction (Ekberg et al., 1996; Nordlund and Reichard, 2006; Cotruvo and Stubbe, 2011). Furthermore, all class I RNRs requires the presence of oxygen for the generation of a radical under aerobic conditions.

#### Class II

Class II RNRs comprise a single α-chain polypeptide encoded by a single *nrdJ* gene. NrdJ harbors the active center and allosteric sites of the enzyme. This RNR class uses S-adenosylcobalamine (AdoCob) to generate the cysteinyl radical that substitutes the class I small protein (ß subunit) (Tamao and Blakley, 1973; Licht et al., 1996). This enzymatic reaction does not require oxygen, as this RNR class is completely oxygen independent. Class II RNRs harbor an allosteric specificity site, but lack the allosteric activity site, similar to class Ib RNRs (Eliasson et al., 1999; Larsson et al., 2010). An exceptional class II RNR has been identified in *P. aeruginosa*, and this enzyme differs from all hitherto known class II RNRs, as this enzyme is split and encoded by two consecutive open reading frames, namely *nrdJa* and *nrdJb*, separated by 16 bp (Torrents et al., 2005).

#### Class III

Class III RNRs comprise two homodimeric proteins encoded by *nrdD* and *nrdG* genes. NrdD is the large enzymatic catalytic subunit, harboring the active site and the two allosteric regulation sites, respectively determining substrate specificity and activity. Furthermore, the NrdG protein (known as activase) is responsible for generating the radical (Sun et al., 1993, 1995). Class III RNRs require the binding of S-adenosylmethionine (SAM) to a 4Fe-4S metal center located in the NrdG protein for radical formation (Eliasson et al., 1990). This interaction generates an extremely oxygen sensitive glycyl radical at the C-terminal domain of the NrdD protein (King and Reichard, 1995). Owing to the high sensitivity of the glycyl radical and the potential metal center oxidation in the presence of oxygen, this RNR class is only active under anaerobic conditions.

Experiments on the *Lactococcus lactis* class III RNR have shown that NrdD alone catalyzes the reduction of NTPs (Torrents et al., 2001). After activation of NrdD, NrdG was no longer required and dissociated from the complex. Activated NrdD protein can catalyze several rounds of NTP reduction in the presence of formate as an electron donor, with ATP as the allosteric effector and requiring Mg^2+^ and K^+^ to stimulate the reaction (Torrents et al., 2001). This enzymatic reaction is similar to that of the *E. coli* pyruvate formate lyase (PFL) enzyme system (Kessler and Knappe, 1996), in which PFL is the catalytic subunit component and PFL-activase initiates the system.

### Distribution

The distribution of the different RNR classes among the different organisms on Earth is remarkable. Several organisms have been sequenced and some encode different functionally redundant RNR classes. Moreover, a combination of different RNR classes can be encoded in the same organism. This occurrence is complex, and the RNR class is not always associated with the life style and phylogeny of the organism (Torrents et al., 2000, 2002; Lundin et al., 2009, 2010). Table 2 provides a short list of the RNR occurrence among eukaryotes, archaea, and eubacteria. A complete list of the distribution of all RNR classes is provided in the RNR database (RNRdb) (Lundin et al., 2009).

**Table 2:** **The distribution of RNR in the three domains of life**.

**EUKARYOTA**					
*Homo sapiens* (humans)	Ia				
*Sacharomyces cerevisiae* (yeast)	Ia				
*Arabidopsis thaliana* (plant)	Ia				
*Gibberella zeae*	Ia				III
*Trichomonas vaginalis*				II	
**ARCHAEA**					
*Pyrococcus furiosus*				II	III
*Sulfolobus islandicus*				II	
*Natromonas pharaonis*	Ia			II	
**EUBACTERIA**					
*Bacillus subtilis*		Ib			
*Bacillus anthracis*		Ib			III
*Listeria monocytogenes*	Ia				III
*Clostridium tetani*				II	III
*Lactobacillus casei*		Ib		II	III
*Staphylococcus aureus*		Ib			III
*Mycoplasma mobile*				II	
*Actinomyces urogenitalis*		Ib			III
*Mycobacterium tuberculosis*		Ib		II	
*Porphyromonas gingivalis*				II	III
*Nostoc azollae*				II	
*Escherichia coli*	Ia	Ib			III
*Pseudomonas aeruginosa*	Ia			II	III
*Vibrio cholerae*	Ia				III
*Salmonella enterica*	Ia	Ib			III
*Aeromonas hydrophila*	Ia			II	III
*Burkholderia cenocepacea*	Ia			II	
*Chamydia trachomatis*			Ic		
*Tropheryma whipplei*			Ic		

Complex eukaryotic organisms only encode class Ia RNRs, thus limiting their survival in aerobic environments. Nevertheless, some unicellular algae (e.g., *Euglena gracilis*) also encode an active class II RNR (Torrents et al., 2006a), but the number of eukaryotic organisms with this atypical RNR occurrence is low. Furthermore, some fungi genomes show class III RNR sequences (e.g., *Gibberella zeae*), but surprisingly, these enzymes are atypical compared with other class III RNRs, and further studies are needed to elucidate whether this RNR class is functional (Torrents et al., 2008).

Any potential RNR combination can be identified in eubacteria and archaea. Some bacteria encode only one RNR class, such as *Bacillus subtillis* (class Ib), while other bacteria encode two RNR classes, such as *Staphylococcus aureus* (class Ib and III) (Kirdis et al., 2007), or a higher level of complexity, such as in *Pseudomonas aeruginosa*, which encodes three different RNR classes (Ia, II, and III) (Jordan et al., 1999; Torrents et al., 2005; Sjöberg and Torrents, 2011).

**Figure 3:**
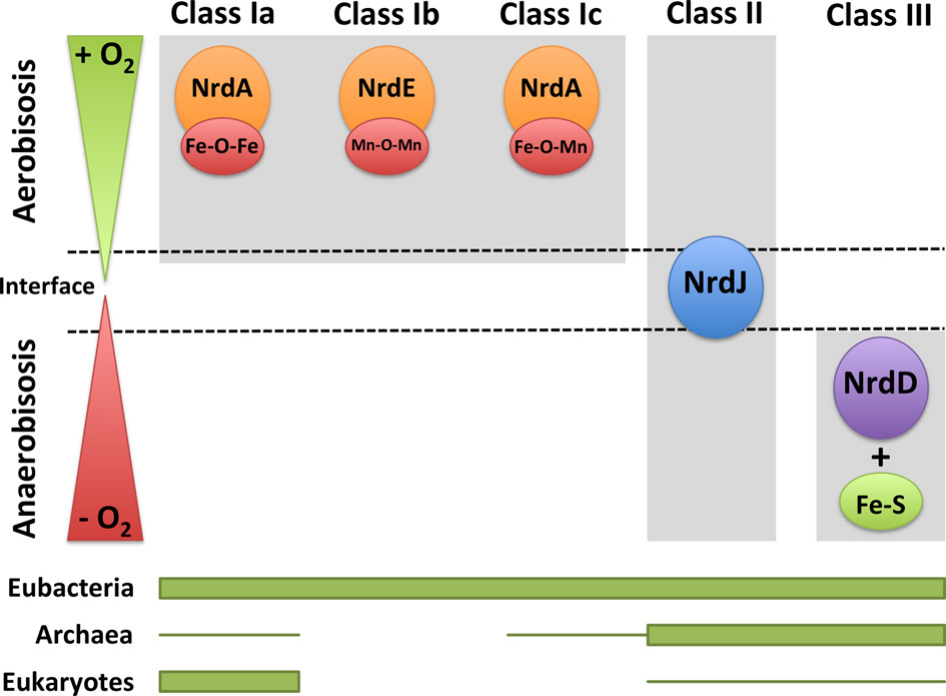
**RNRs as survival markers under aerobic or anaerobic environments**. RNR classes can be activated under different environmental conditions in which one organism survives depending on the oxygen availability (aerobic, anaerobic, and in the interface). The distribution of each RNR class in the three domains of life (eubacteria, archaea, and eukaryotes) is shown. The thicker line represents higher RNR class occurrence, and the thinner line corresponds to the occurrence in only a few organisms.

Ribonucleotide reductases function under a range of different environmental conditions; indeed, the type of RNR class will impact the adaptability of microorganisms to different environmental conditions. Bacteria encoding more than one RNR class can survive in a wide range of ecological niches (Figure 3). For example, class II or III RNRs facilitate survival under anaerobic conditions. Furthermore, the presence of class II RNRs facilitates the survival of bacteria in the transition from aerobic to anaerobic environments, or vice versa, and also at the interface of both oxygenic conditions. Indeed, the proper functionality of class II RNRs depends on S-adenosylcobalamine (vitamin B_12_) availability, as previously observed in *P. aeruginosa*; thus, vitamin B_12_ is a limiting cofactor for the activity of this RNR class (Torrents et al., 2005; Sjöberg and Torrents, 2011). Class I RNRs, and no other RNR classes, require an aerobic environment, as these enzymes are oxygen dependent. In addition, bacteria encoding both class Ia and Ib RNRs facilitates survival under environmental conditions in which iron is limiting, as class Ib RNRs substitute iron from manganese in the metal center (Martin and Imlay, 2011). Moreover, it has been shown that class Ib RNRs are highly expressed and essential for *E. coli* biofilm formation, under nutrient-limited conditions (Cendra et al., 2012) or high oxidative stress (Monje-Casas et al., 2001), indicating the use of class Ib RNRs under special growth conditions.

### RNR evolution

The maintenance of life on Earth depends on the ability to reproduce. Reproduction requires an accurate and stable storage system for the genetic information in all organisms, including viruses. It has been recently suggested that the RNA molecule, with auto-replicative capacity, is the primary primitive molecule for the genetic information storage. Despite the wide acceptance of this idea, there are arguments against the concept of an RNA world that cannot be underestimated.

The transition from an RNA to a DNA-protein world, which currently exists, implies the existence of ribosome-based translation, a genetic code and the replacement of RNA with DNA molecule as the genetic material. The results from recent studies concerning the characterization of RNRs and the current knowledge of the evolution of organisms on Earth could help determine which type of RNR enzyme is most similar to the ancestral RNR (ur-RNR), and this information will lead to a better understanding of the transition from a RNA to a DNA world (Reichard, 1993; Stubbe, 2000; Stubbe et al., 2001).

Today, three different RNR classes have been described, with little apparent similarity between them in terms of primary protein sequence (approximately 10–20% similarity). Thus, it could be assumed that each RNR class appeared independently from each other over time. But, surprisingly, there is a great similarity the reaction mechanism, allosteric regulation and three-dimensional structure (tertiary structure) of these enzymes, suggesting a potential common origin (Logan et al., 1999; Sintchak et al., 2002; Torrents et al., 2002).

Based on the geological history of the Earth and trace element content of the sediments, early stages of life originated in an anaerobic environment (Large et al., 2014). Under this restrictive condition, class III RNRs were, most likely, the primitive form from which all other RNR enzymes were derived, and this enzyme class is might be the ancestral RNR (ur-reductase). Moreover, the enzymatic activation of class III RNR requires S-adenosylmethionine (SAM), one of the most ancestral molecules, with few steps required for its biosynthesis (Frey et al., 2008). NrdG protein harbors an Fe-S cluster for glycyl radical generation, and this cluster is considered among the most ancient, ubiquitous, and functionally diverse among biological prosthetic groups (Beinert et al., 1997). Furthermore, this RNR class uses formate as the external reductant, which is a simple molecule compared with the electron donors used for other RNR classes (thioredoxin, glutaredoxin, or NrdH). Class II RNRs also function under anaerobic conditions, but other factors determine whether these enzymes act as ur-reductases. Class II RNRs use S-adenosylcobalamine (AdoCob) for radical generation, which is more structurally complex than SAM. However, other studies have suggested that class II RNRs represent an ancient ur-RNR class (Poole et al., 2002). Certainly, the appearance of oxygen on Earth was key to this evolutionary process, culminating in the existence of the different RNR classes observed today.

### Genetic regulation

An unbalanced pool of dNTPs leads to an increase in the mutation rate and the loss of DNA replication fidelity (Wheeler et al., 2005; Mathews, 2006). Thus, RNR activity must be highly regulated at either the enzymatic activity level through allosteric regulation, as previously described, or at the transcriptional level.

Most RNRs, in both microorganisms and eukaryotic cells, are transcriptionally regulated depending on the cell cycle, as DNA replication primarily occurs during cell division where a high concentration of different dNTPs is needed. Here, the transcriptional regulation of RNRs in microorganisms, particularly eubacteria, is discussed. Several other studies have previously addressed eukaryotic RNR transcriptional regulation (Hakansson et al., 2006; Nordlund and Reichard, 2006; Thelander, 2007).

Transcriptional regulation is complex in organisms encoding more than one RNR class, as this process is essential to achieve adequate coordination of the expression of the different *nrd* genes to ensure proper enzyme concentration and provide balanced dNTPs levels. For example, in *Escherichia coli*, class Ia RNRs are active during growth under laboratory conditions, while class III enzymes are active under anaerobic conditions (Boston and Atlung, 2003; Roca et al., 2008a) and class Ib RNRs are important during growth in iron-deficient medium (Martin and Imlay, 2011) and biofilm formation (Cendra et al., 2012). However, *Pseudomonas aeruginosa* also express class Ia RNRs under laboratory growth conditions (Jordan et al., 1999; Torrents et al., 2006b) and class II and III RNRs are expressed during infection (Sjöberg and Torrents, 2011).

Several transcription factors (activators and inhibitors) have been implicated for regulation the expression of each RNR class during bacterial growth under certain environmental conditions. For example IciA, FIS, and DnaA are transcriptional activators for *E. coli* class Ia RNRs, while H-NS and NrdR act as a transcriptional repressors (Torrents et al., 2007; Cendra et al., 2012, 2013). Class Ib RNRs are transcriptionally activated through FUR (Vassinova and Kozyrev, 2000; McHugh et al., 2003). Moreover, class III RNRs are transcriptionally regulated through the FNR protein (Boston and Atlung, 2003; Roca et al., 2008a).

In 2007 and 2008, comprehensive reviews were published (Herrick and Sclavi, 2007; Torrents et al., 2008) concerning the transcriptional regulation of certain RNR classes in *E. coli* and other bacteria; however, in this chapter, the focus will be on the recently described transcriptional regulator NrdR, implicated in the regulation of all three RNR classes.

#### NrdR—a global ribonucleotide reductase regulator

Recent publications have described the transcriptional regulation of different RNR classes; however, little is known about how the expression of different RNRs might be coordinated in microorganisms encoding several RNR classes. A novel transcriptional factor, NrdR, has recently been implicated in the regulation of all three RNR classes through binding to conserved NrdR boxes in the promoter regions of almost all genes encoding RNRs (Grinberg et al., 2006; Torrents et al., 2007; Mowa et al., 2009; Panosa et al., 2010; Case et al., 2011). All currently known eubacteria encode and *nrdR* gene except that *Rickettsia, Helicobacter*, and *Desulfovibrio*.

The results obtained from a recent study conducted at Tel Aviv University (Israel) indicated a role for NrdR in the transcriptional regulation of different RNR genes encoded in a single organism. This group was the first to identify an open reading frame in *Streptomyces coelicolor*, encoding NrdR, a protein that binds to the promoter regions of class Ia and II RNRs (Borovok et al., 2004). Subsequently, a computational study comparing all RNR promoter regions at the genomic level revealed a highly conserved palindromic sequence, named the NrdR-box, with the consensus sequence acaCwAtATaTwGtg (Rodionov and Gelfand, 2005). This NrdR-box has since been identified in eubacterial genomes and is absent in archaea and eukaryotes. Typically two copies of the NrdR-box are consistently detected in the promoter region of different *nrd* genes, with specific spacing corresponding to an integral number of turns in the double DNA helix.

**Figure 4:**
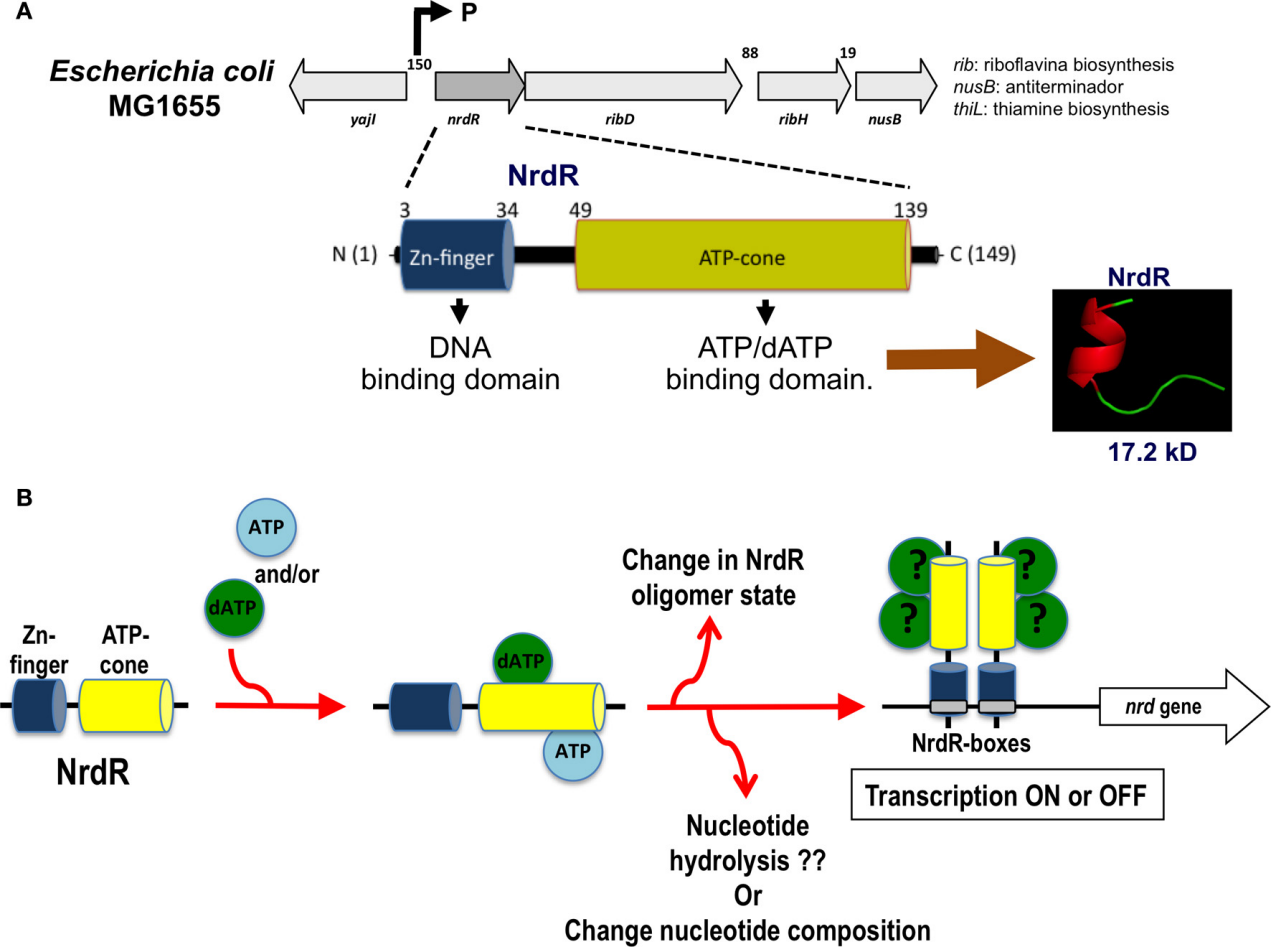
***nrdR* operon organization and hypothetical mechanism of transcription regulation by NrdR. (A)** Structure of the *nrdR* operon and functional protein domains for the NrdR transcriptional regulator. **(B)** Hypothetical mechanism for the transcriptional regulation of the *nrd* gene through NrdR. Depending on which nucleotide is bound and the oligomerization state of the NrdR, this transcription factor can modulate the expression of the *nrd* genes through binding to the specific NrdR-box.

Currently, NrdR has been classified as a member of a highly conserved family of proteins confined exclusively to prokaryotes, eubacteria, and some archaea (Lundin et al., 2009). NrdR is a 150–200-amino acid protein harboring two protein domains: an N-terminal zinc finger like DNA-binding domain and a C-terminal ATP-cone domain that binds nucleotides (Figure 4A). The N-terminus contains a zinc ribbon motif for binding to the upstream regulatory regions of the *nrd* genes (NrdR-box). The ATP domain is similar to the allosteric activity domain observed in some RNRs. The *Streptomyces coelicolor* NrdR protein is the best characterized (Grinberg et al., 2006, 2009). Studies have demonstrated that the ATP-cone domain alone is essential for nucleotide binding and important for the correct three-dimensional NrdR structure and DNA binding to the NrdR-box. One hypothesis is that NrdR regulates RNR transcription by acting as ATP/dATP-dependent regulator that controls the oligomeric state of NrdR. Thus, a native NrdR oligomer, likely comprising eight subunits, binds ATP or dATP, and the zing finger domain is free to bind to the DNA target site to repress RNR gene expression. When DNA replicates, the cellular levels of dNTPs are decreased, NrdR is depleted of dATP, and the ATP-cone domain undergoes a change from an oligomeric state to a dimeric protein, abolishing its DNA binding activity and increasing *nrd* transcription (Figure 4B).

A recent study reported a more complex mechanism of NrdRs nucleotide binding through controlled complex allosteric mechanisms. A model has been proposed, in which NrdR selectively binds nucleotide triphosphates, which are subsequently hydrolyzed to monophosphates to regulate the NrdR oligomeric state and DNA binding (McKethan and Spiro, 2013).

Hence, NrdR might be a transcriptional regulator with complex cooperative and allosterically regulated nucleotide binding mechanism that finely tunes the expression of the ribonucleotide reductase gene in response to cellular nucleotides.

### Ribonucleotide reductase as a biomedical target

RNRs are essential enzymes for all organisms, as these proteins provide the dNTPs needed for chromosome replication and DNA damage repair. The contribution of RNR to these key reactions makes this enzyme a perfect target for the design of compounds that inhibit cell growth in either cells with altered cell cycles (cancer cells) or during viral, bacterial or protozoan infections (Torrents et al., 2008).

**Figure 5:**
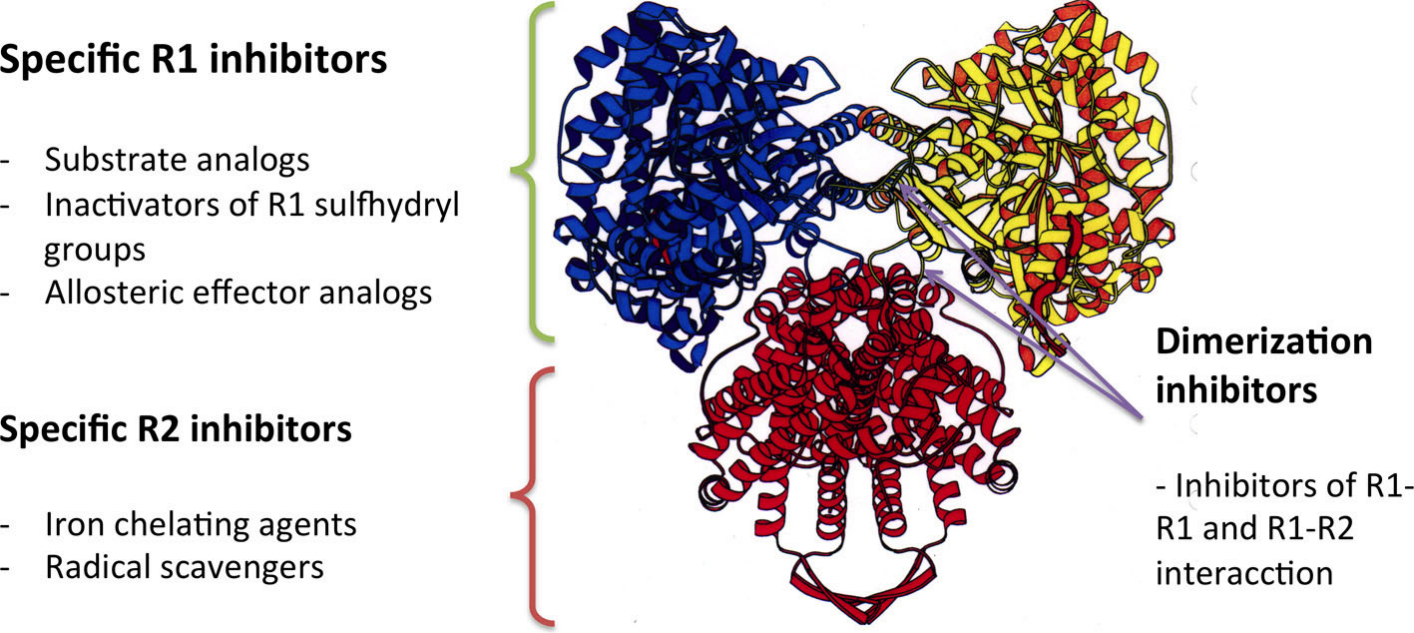
**The mode of action of different RNR inhibitors**.

RNRs are complex enzymes, and several inhibitors of these enzymes have been described and classified according to their mode of action (Figure 5). The first group of inhibitors identified interacted directly with the catalytic subunit (R1), alternating enzymatic activity through the irreversible binding to the active site of the enzyme (substrate analogs or inactivators of sulfhydryl groups) or to allosteric binding sites. The inhibitors in the second group interact with the activator subunit (ß) of class I RNRs, and have been identified as either chelating agents of the dinuclear iron center or radical scavengers. The third group of inhibitors comprises antisense inhibitors, which are antisense RNA molecules that bind to mRNA encoding the components of the enzyme. These antisense inhibitors have shown promising results in some cancers. Moreover, there are inhibitors that prevent the dimerization of the enzyme components, which are peptides that mimic the interaction between the catalytic and activator subunit (R1-R2) or the interactions between the catalytic subunits themselves (R1-R1) (Torrents et al., 2008; Wijerathna et al., 2011; Basu and Sinha, 2012; Bhave et al., 2013; Liu et al., 2013; Zhou et al., 2013).

### Conflict of interest statement

The author declares that the research was conducted in the absence of any commercial or financial relationships that could be construed as a potential conflict of interest.
